# Gas6 Stimulates Angiogenesis of Human Retinal Endothelial Cells and of Zebrafish Embryos via ERK1/2 Signaling

**DOI:** 10.1371/journal.pone.0083901

**Published:** 2014-01-07

**Authors:** Young Sook Kim, Seung-Hyun Jung, Dong Ho Jung, So-Jin Choi, Yu-Ri Lee, Jin Sook Kim

**Affiliations:** Korean Medicine-Based Herbal Drug Development Group, Herbal Medicine Research Division, Korea Institute of Oriental Medicine (KIOM), Daejeon, Republic of Korea; University Heart Centre Freiburg, Germany

## Abstract

**Aim:**

To determine if growth arrest-specific 6 (Gas6) plays an important role in the regulation of angiogenesis in human retinal microvascular endothelial cells (HRMECs) and in vessel development of zebrafish.

**Methods:**

Proliferation, wound-healing cell migration, and tube formation were measured in HRMECs treated with recombinant human Gas6 (rhGas6). Sprague-Dawley rat aortas in Matrigels were treated with rhGas6, and microvessel sprouting emanating from arterial rings was analyzed. Transgenic zebrafish embryos (flk:GFP) were microinjected with rhGas6 at 50 hours post-fertilization (hpf), and ectopic sprouting of subintestinal vessels (SIVs) was observed under a confocal microscope. Morpholino oligonucleotides (MOs) were microinjected to knockdown *gas6* in zebrafish embryos, and intersegmental vessel impairment was observed. The effect of the extracellular signal-regulated kinase (ERK1/2) inhibitor on the migration of HRMECs and on vessel development in zebrafish embryos was tested.

**Results:**

rhGas6 stimulated proliferation, migration, and tube formation in HRMECs in a dose-dependent manner. In rat aortas, rhGas6 induced vessel outgrowth, and the sprouting length was longer than that of controls. The rhGas6-microinjected zebrafish embryos had significantly increased vessel outgrowth in the SIVs. Recombinant human vascular endothelial growth factor (rhVEGF) served as a positive control. Knockdown of *gas6* inhibited angiogenesis in the developing vessels of zebrafish. The ERK1/2 inhibitor inhibited HRMEC migration and intersegmental vessel formation in zebrafish embryos.

**Conclusions/Interpretations:**

These data suggest that Gas6 plays a pivotal role in proliferation, migration, and sprouting of angiogenic endothelial cells in the retina and in zebrafish embryos. Furthermore, Gas6 induced angiogenic processes are induced via phosphorylation of ERK1/2.

## Introduction

Angiogenesis is the growth of new vessels from the existing vasculature in various organs. Angiogenic processes play a critical role in the physiologic conditions of cancer, heart diseases, atherosclerosis, and various eye diseases [1]. The formation of abnormal neovascularization and the growth and spread of vessels in the eye are the most common causes of blindness. Such alterations occur in age-related macular degeneration, central retinal vein occlusion, and diabetic retinopathy. Angiogenic factors such as vascular endothelial growth factor (VEGF), platelet-derived growth factor (PDGF), fibroblast growth factor (FGF), insulin-like growth factor (IGF), and angiopoietin have been implicated in dysregulation of growth, migration, adhesion, and differentiation of retinal vascular cells in retinal vascular diseases [2]–[4]. Nonetheless, the mechanism underlying pathophysiological neovascularization in the retina remains poorly understood.

Growth arrest-specific 6 (Gas6) is a vitamin K dependent protein discovered through the screening of genes upregulated in growth-arrested embryonic mouse fibroblasts [5]. Gas6 is a ligand for the tyrosine protein kinase receptors Axl, Mer, and Tyro3, which have been implicated in vascular homeostasis, cell growth, survival, and platelet thrombus formation [6]. Thus far, Axl and Gas6 signaling is implicated in cell proliferation, migration, and invasion during tumor angiogenesis, as well as in diabetic nephropathy [7], [8]. Gas6 is a novel growth factor for kidney mesangial cells, and is posttranslationally activated by C-carboxylation in the presence of vitamin K. In diabetic nephropathy, streptozotocin-treated Gas6 knockout mice exhibit less pronounced glomerular hypertrophy and glycoxidized low-density lipoprotein increase in mouse mesangial cells [7], [9]. However, nothing is known regarding the relationship between Gas6 and neovascularization in the retina and in developing zebrafish embryos.

In the present study, we investigated the effect of Gas6 on cell proliferation and migration under normal growth conditions of retinal microvascular endothelial cells (HRMECs) and the effect of Gas6 in developing zebrafish embryos. We provide evidence that Gas6 plays a crucial role in angiogenesis via regulating extracellular signal-regulated kinase (ERK1/2) phosphorylation in HRMECs and zebrafish. Our data reveal an unexpected function of Gas6 as an angiogenic factor that promotes cell survival and proliferation.

## Materials and Methods

### Cell culture

HRMECs (Cat. No. ACBRI 181) were purchased from Cell Systems (Kirkland, WA) and used at passages 3–7. Cells were grown in CSC complete medium (CS-4ZO-500; Cell Systems) containing Bac-Off® (antibiotic). Cultures were maintained at 37°C in a humidified 95% air/5% CO_2_ atmosphere. Quiescence was induced by incubating the cells in CSC complete serum-free medium for 24 h. Cells were then used for the experiments, unless otherwise indicated.

### Zebrafish and angiogenesis assays

Adult zebrafish were maintained under standard conditions at 28.5°C with a 14 h light/10 h dark cycle. Embryos were obtained from crosses between flk:GFP transgenic fish and raised in embryonic water. All experimental protocols for animal care and use were approved by the local ethical board (Korea Institute of Oriental Medicine Animal Care and Use Committee), and animal husbandry and procedures were performed according to institutional guidelines. For angiogenesis assays, dechorionated anesthetized zebrafish embryos in egg water, with Tricaine (0.016% MS222, Sigma-Aldrich, St. Louis, MO) added, were placed on agarose-modified dishes and microinjected (∼2 nl) with recombinant human Gas6 (300 ng/µl, rhGas6; Cat. No. 885-GS-050, R&D Systems, Minneapolis, MN) or recombinant human VEGF (5 ng/µl, rhVEGF, 293-VE-001MG/CF, R&D Systems) into the perivitelline space at 50 h post-fertilization (hpf) and maintained under standard conditions at 28.5°C, as previously described [10], [11]. Fluorescence images were collected using a confocal microscope (Olympus, FV10i, Center Valley, PA) at 80 hpf. Embryonic stages were determined by hpf.

### Proliferation assay

Cell proliferation was measured using the 3-(4,5-dimethyl thiazol-2-yl)-2,5-diphenyl tetrazolium bromide (MTT) assay kit (Cell Proliferation Kit I, Cat. No. 11 465 007 001, Roche, Nutley, NJ). Cells were plated (1×10^4^ cells/well) in quadruplicate into 96-well plates with various doses of rhGas6 and rhVEGF. Cell viability was measured at 24 h and 48 h after incubation, and 10 µl MTT solution was added to the wells and incubated for 4 h. After incubation at 37°C, 100 µl solubilization solution was added into each well and incubated for 24 h. Absorbance was measured in a microplate autoreader (BIO-TEK, Synergy HT, Winooski, VT) at 550 nm. All experiments were repeated at least three times.

### Wound-healing cell migration assay

HRMECs were plated at 1×10^5^ cells/well on a 12-well plate in normal culture medium and allowed to reach 80–90% confluency. An injury line with a width of 0.6∼1 mm was made with a sterile pipette tip and cells were rinsed with phosphate-buffered saline (PBS). Fresh culture medium containing rhGas6 or rhVEGF was placed into the wells, and the cells were incubated for 6 h. To specifically determine the role of Gas6 in migration, cells were pretreated with depletion media containing 0.5% bovine serum albumin (BSA) with or without warfarin (1 µM, Sigma-Aldrich). Cell migration was monitored by visual examination using an inverted microscope (BX51 Olympus), as previously described [12].

### Rat aortic ring-sprouting assay

Aortas were harvested from 6-week-old Sprague-Dawley rats and immediately placed on ice in PBS supplemented with 1% fetal bovine serum. Plates (24-well) were coated with 200 µl Matrigel (Cultrex®, Trevigen Inc., Gaithersburg, MD). After polymerization, the rings were carefully placed in the wells with fine micro-dissecting forceps and sealed in place with an overlay of 100 µl Matrigel. rhGas6 (200 and 400 ng/ml) or rhVEGF (20 ng/ml) was added to the wells in a final volume of 1 ml serum-free medium and incubated at 37°C in 5% CO_2_. rhVEGF was used as a positive control. After 5 days, images of the aortic rings were obtained using an inverted microscope (BX51 Olympus). Sprouting length was quantified as mean maximal sprout length from the perimeter of the aortic ring to the most distal tip of the angiogenic sprout in four quadrants of each aortic ring. Experiments were performed in triplicate.

### Tube formation assay

Briefly, 24-well tissue culture plates were coated with 200 µl basement membrane-like extract (BME/Matrigel) and incubated for 30 min at 37°C. HRMECs were seeded at 1×10^6^ cells/ml and treated with serum-free medium containing the vehicle (PBS) or 400 ng/ml rhGas6 or 20 ng/ml rhVEGF. The cells were cultured for 9 h at 37°C in 5% CO_2_. The tube networks were stained with calcein AM (Invitrogen Life Sciences, Grand Island, NY) and observed with an inverted fluorescent microscope (BX51 Olympus). Images were captured using a digital camera (DP 70 Olympus). Four fields per well were captured for quantitative analysis. Sprout length was measured under an inverted microscope at 100× magnification and quantified. The digitized images were imported into Image J software. The experiments were independently repeated three times.

### Western blot analysis

Aliquots of protein were treated with Laemmli sample buffer (Bio-Rad, Hercules, CA), heated to 100°C for 5 min, and electrophoresed with 20 µg protein/lane on a denaturing sodium dodecyl sulfate polyacrylamide gel. Proteins were then transferred to a nitrocellulose membrane (Whatman, GmbH, Hahnestr, Germany) using a tank blotting apparatus (Bio-Rad). Membranes were probed with 1∶1000 dilutions of polyclonal antibodies against phosphorylated ERK1/2, p38, and c-Jun N-terminal kinase (JNK) (Cell Signaling Technology, Danvers, MA). The membrane was washed and incubated with a horseradish peroxidase-linked goat anti-rabbit IgG (Santa Cruz Biotechnology, Santa Cruz, CA). After washing the membranes three times, the signals were detected with a WEST-one™ enhanced chemiluminescence solution (GenScript, Piscataway, NJ) using Fujifilm LAS-3000 (LAS-3000, Fuji Photo, Tokyo, Japan).

### Immunofluorescence staining

To determine the expression levels of pERK1/2 in HRMECs, Gas6 treated HRMECs were fixed in 2% paraformaldehyde. Cells were permeabilized and blocked with 0.1% Triton X-100/PBS containing 2% BSA and 0.5% normal serum to reduce the nonspecific adherence of antibodies. Cells were incubated in primary anti-pERK1/2 at a dilution of 1∶1000 for 1 h at 37°C in a humidified chamber. After incubation with primary antibody, the cells were rinsed and incubated with anti-rabbit IgG -FITC (Santa Cruz Biotechnology). Cell nuclei were stained with Hoechst stain (blue). After washing with PBS and treating with mounting solution (Wako, Kumamoto, Japan), cells were observed under an Olympus IX-81 fluorescence microscope using a 20× objective.

### Knockdown by *gas6* morpholino oligonucleotide in zebrafish embryos

Transcript and genome sequence data of *gas6* reported are available in the NCBI and ensemble database under accession numbers NM199978, ENSDARG00000007804 (zebrafish) and NM000820, ENSG00000183087 (human). Zebrafish show 66% homology with human gas6. To knockdown in zebrafish embryos, morpholino antisense oligonucleotides (MOs) for *gas6* EX5-MO (exon 5 splice donor site; 5′- GCT GAC GGT GTG TTT TTA CCG TTC T -3′), *gas6* EX7-MO (exon7 splice donor site; 5′- TGG TGT TGC TGA AGA CTG ACC TAC A -3′), and standard Ctrl-MO (control morpholino, 5′- CCT CTT ACC TCA GTT ACA ATT TAT A-3′) were designed and synthesized by Gene-Tools, LLC (Corvallis, OR). MOs were resuspended in 1× Danieau's buffer [58 mM NaCl, 0.7 mM KCl, 0.4 mM MgSO_4_, 0.6 mM Ca(NO_3_)_2_, 5 mM HEPES, pH 7.6] with 0.1% phenol red, and microinjected into embryos at the 1–4 cell stage (1–5 ng/embryo). Injected embryos were incubated until the indicated stage and photographed using a fluorescent dissecting microscope (Olympus SZX16). Efficacy of morpholinos on the pre-mRNA splicing was evaluated using RT-PCR with forward primer designed to exon 1 (5′- GCC ATG AGG GAG CTG GTG TGG AGC-3′) in conjunction with reverse primer designed to exon 8 (5′- CAG ACG ACC GTC ACA GAA GCA GCG-3′). Total RNA from embryos was extracted using a standard protocol with TRIzol reagent (Invitrogen) and reverse transcribed into cDNA using M-MLV reverse transcriptase. The RT product was used as a template for PCR amplification of *gas6* and *β-actin* using the following cycles with 5 pmol primers and Taq DNA polymerase (Elpis, Pusan, Korea): 95°C for 30 s, 56°C for 30 s, and 68°C for 50 s for 30 cycles.

### Chemical treatment of zebrafish embryos

Transgenic (flk:GFP) zebrafish embryos were treated with SB203580 (p38 MAPK inhibitor, Calbiochem, Billerica, MA), PD98059 (p42/44 MAPK inhibitor, Calbiochem) and U0126 (MEK/ERK inhibitor, Cell Signaling Technology) that were diluted in dimethyl sulfoxide (DMSO). The embryos were incubated with 40 µM SB203580, 20 µM PD98059, and 40 µM U0126 at 12 hpf and observed using a confocal microscope (Olympus, FV10i) at 30 hpf.

### Whole-mount immunostaining

Whole-mount immunostaining was carried out as previously described [13]. Briefly, transgenic (flk:GFP) zebrafish embryos were treated with U0126 (MEK/ERK inhibitor, Cell Signaling Technology). The embryos were incubated with 40 µM U0126 for 14 h, fixed at 26 hpf, and stained with anti-phospho-ERK1/2 antibody (1∶500, Cell Signaling Technology). For fluorescent detection of the antibody, Alexa Fluor 568 anti-rabbit conjugate was used (1∶500, Molecular Probes, Grand Island, NY). All stained embryos were mounted with glycerol and photographed on an Olympus FV10i confocal microscope and their expression was analyzed by Image J software.

### shRNA knockdown and quantitative real-time PCR (QRT-PCR)

HRMECs were cultured to 80% confluence and transfected with Axl, Mer, and Tyro3 shRNA. Transfection of plasmids into HRMEC with the Neon Transfection System (Invitrogen) was performed according to the manufacturer's protocol with two pulses of 1400 V and 20 ms. After transfection for 24 h, the cells were collected for real-time PCR. Briefly, total RNA from cells was extracted using a standard protocol with TRIzol reagent (Invitrogen) and first-strand complementary DNA was synthesized in a 20 µl reaction volume using 0.5 µg of total RNA. Reverse transcript products were obtained using a TaKaRa PrimeScriptTM 1st Strand cDNA Synthesis kit (TaKaRa, Mountain View, CA) followed by QRT-PCR on a iQ5 Continuous Fluorescence Detector System (Bio-Rad). The PCR reactions contained 250 nM of primers, 1 µl cDNA (5 ng), and 10 µl 2X SYBR-green Realtime PCR Master Mix (SYBR Premix Ex Taq TM, TaKaRa) in a total volume of 20 µl with at least two independent biological sample repeats and four technical assay repeats. Error bars were generated from the standard deviation (calculated by the Bio-Rad iQ5 Continuous Fluorescence Detector System software). Reactions without template and/or enzyme were used as negative controls.

### Statistical analysis

All experiments were repeated at least three times, and representative data are shown. Data are expressed as the mean ± standard error of the mean (SEM). Differences between groups were analyzed using Student's *t*-test and one-way analysis of variance followed by the Tukey multiple comparison test (PRISM5 software, Graph Pad, La Jolla, CA). Values of *p*<0.05 were considered statistically significant.

## Results

### Gas6 promotes proliferation, migration, and tube formation in HRMECs

**Figure 1:**
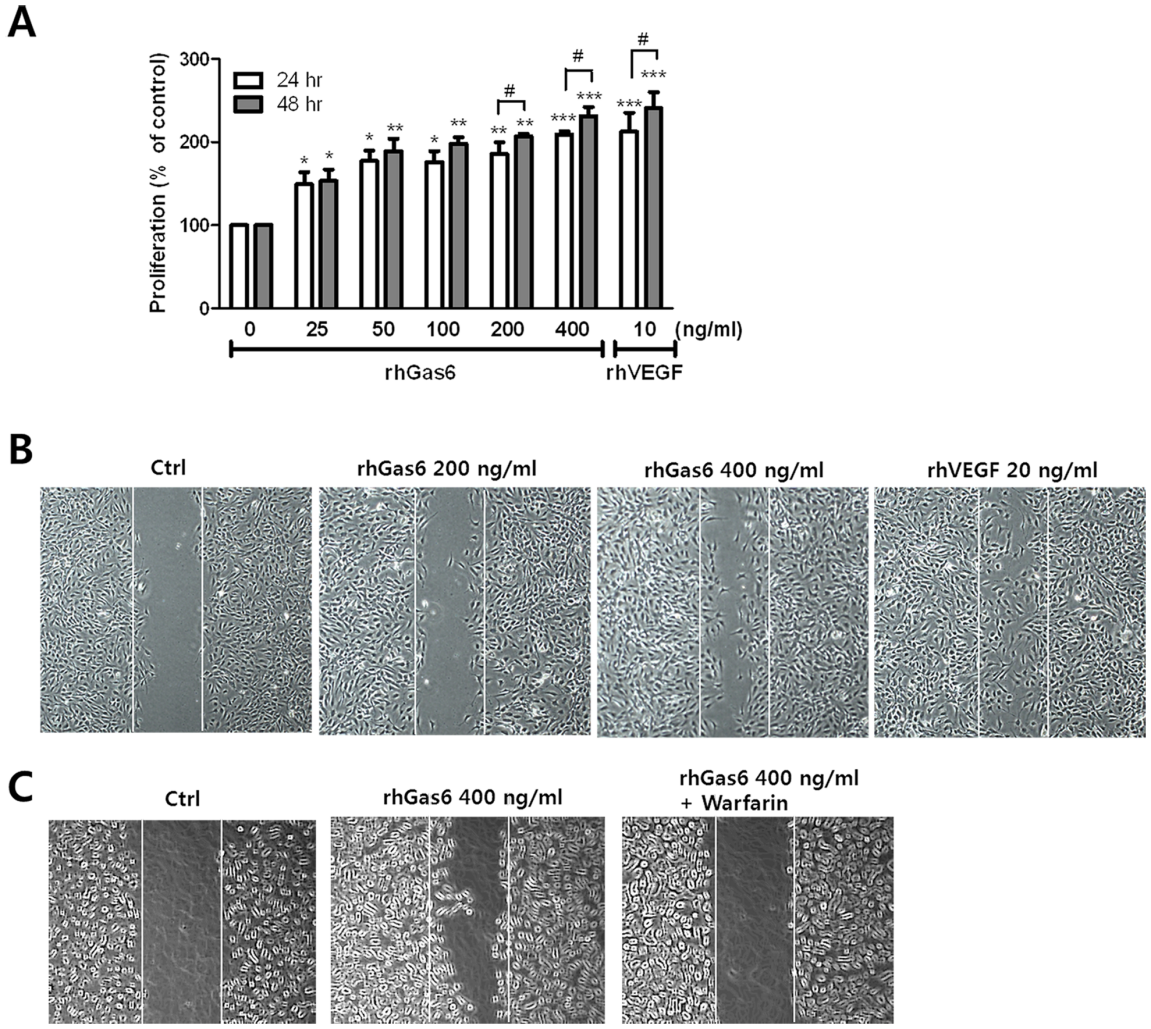
rhGas6 induces proliferation and migration of HRMECs. HRMECs were incubated in the presence of the indicated concentrations of rhGas6 for 24(**A**). Proliferation was determined using the MTT assay, and absorption was analyzed at 550 nm using a microtiter plate reader. The results are presented as the mean ± SEM. (n = 4). ^***^ *p*<0.001, ^**^ *p*<0.01, and ^*^ *p*<0.05 vs. control, # *p*<0.01. (**B**) HRMEC responses to rhGas6 or rhVEGF were determined using a scratch-wound healing assay. Lines indicate the same width of the gap. Representative images are shown at 6 h after generating the scratch. (**C**) Warfarin (1 µM) was preincubated for 30 min prior to rhGas6 addition. Representative images are shown after generating the scratch.

To examine the effect of Gas6 on the proliferation of HRMECs, we performed MTT assays. As shown in Figure 1A, Gas6 plays a role in promoting HRMEC proliferation in a time- and dose-dependent manner. During the angiogenesis process, endothelial cells were stimulated to migrate and proliferate [14]. To evaluate the impact of Gas6 on HRMEC migration, scratch-wound healing assays were performed. For wound healing, HRMECs were serum starved for 24 h, and conditioned media were incubated with 200 and 400 ng/ml rhGas6 or 20 ng/ml rhVEGF as a positive control. Figure 1B shows the effect of Gas6 on the migration of HRMECs compared with a control group. To examine the effect of Gas6 on the migration, we pretreated with warfarin, which inhibited the activation by Gas6 [15]. As shown as Figure 1B, rhGas6 induced the migration in a dose-dependent manner and the induced migration was inhibited by pretreatment with 1 µM warfarin (Figure 1C).

**Figure 2:**
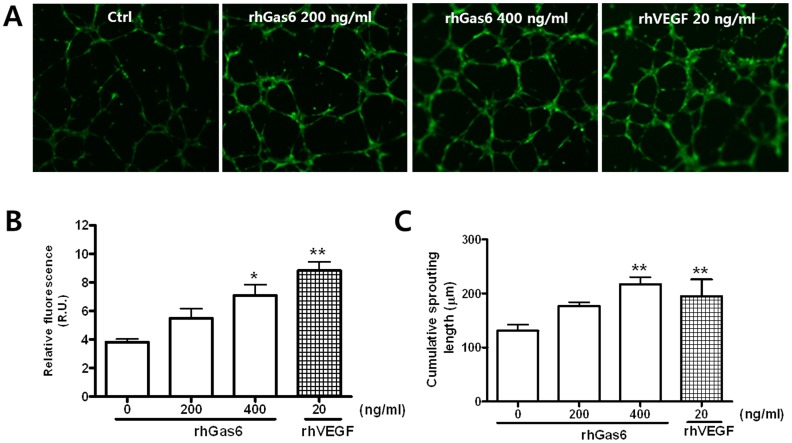
rhGas6 induces tube formation in HRMECs. (**A**) HRMECs were incubated with rhGas6 or rhVEGF. Representative images at 9 h after treatment with rhGas6 or rhVEGF are shown. (**B**) Tube formation by HRMECs on Matrigels was observed by fluorescence microscopy. Relative density was measured using ImageJ software. Data are expressed as mean ± SEM. (n = 4). ^**^ *p*<0.01 and ^*^ *p*<0.05 vs. control. (**C**) Cumulative sprout length was quantified as described in the Materials and Methods. Data are expressed as mean ± SEM. (n = 4). ^**^ *p*<0.01 vs. control.

Next, we examined whether Gas6 could induce tube formation, an endothelial function crucial to angiogenesis. The addition of rhGas6 to the incubation medium led to dose-dependent tube formation (Figure 2). Cumulative sprouting length also increased in a dose-dependent manner in rhGas6 treated cells, with rhVEGF used as a positive control (Figure 2C).

### Gas6 induces vessel outgrowth in the aorta of rats and in zebrafish

**Figure 3:**
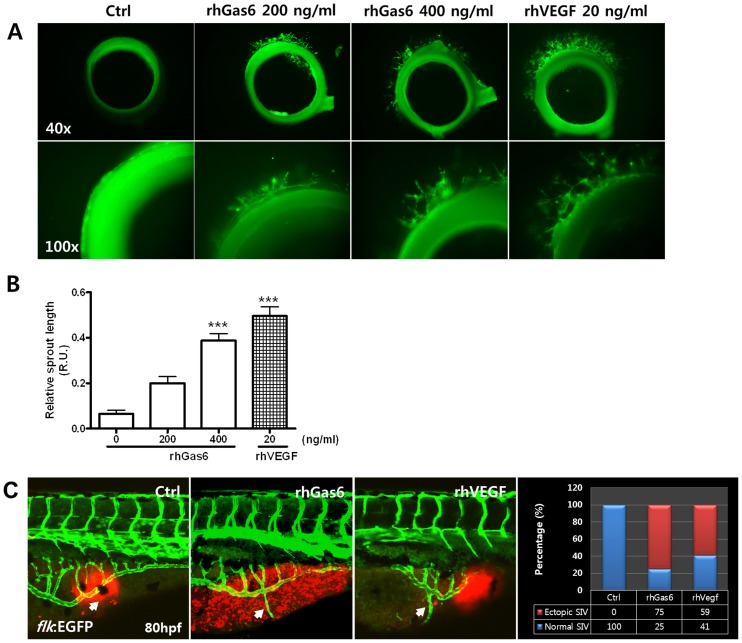
Angiogenic responses are induced by rhGas6 in rat aortic rings and in zebrafish embryos. (**A**) Representative images after 5 day incubation with rhGas6 and rhVEGF. Sprouts from rat aortic rings are shown in the Matrigel control, rhGas6, and rhVEGF after treatment for 5 days, as described in the Materials and Methods. (**B**) Quantification of sprout length from aortic rings revealed increased sprout formation after a 5-day treatment with rhGas6 and rhVEGF in Matrigel. Data are expressed as mean ± SEM. (n = 4). ^***^ *p*<0.001 vs. control. (**C**) Transgenic embryos (flk:GFP) at 50 hpf were injected into the perivitelline space with Texas Red dye (Ctrl), Texas Red dye and rhGas6 (300 ng/µl), or Texas Red dye and rhVEGF (5 ng/µl). After 30 h, embryos were photographed under a confocal microscope. In the controls, the formation of ectopic sprouts (arrow) is never observed, and the injected embryos of the rhGas6- and rhVEGF-treated embryos show ectopic sprouting of subintestinal vessels (SIVs). The experiment was repeated three times (Control, n = 28; rhGas6, n = 24; rhVEGF, n = 24).

To analyze the angiogenic function of Gas6 *ex vivo* and *in vivo*, we used rat aortas [16] and transgenic flk:GFP zebrafish embryos in order to provide angiogenesis model systems [17]. As shown in Figure 3A, rhGas6-treated rat aorta rings showed microvessel outgrowth of endothelial tubules and increased vessel sprouting length. The vessel sprouting length of the aorta was 33% (rhGas6, 200 ng/ml) to 63% (rhGas6, 400 ng/ml) longer than that of control sprouting vessels (Figure 3B).

In zebrafish embryos, rhGas6 or rhVEGF was injected with Texas Red dye as a tracer into the zebrafish embryo yolks at 50 hpf. After 30 h, the extent of subintestinal vessel (SIV) formation was analyzed. Embryos injected with control medium and Texas Red dye served as controls and showed normal SIV development (Figure 3C, left panel). Compared to the control embryos, 75% of rhGas6-injected embryos had significantly increased vessel outgrowth in the SIV, resulting in abnormal SIV patterning (Figure 3C, middle panel, white arrow; right panel). Fifty-nine percent of rhVEGF-injected embryos, used as a positive control, were sufficient to cause the outgrowth of additional vessels, and similar results were observed with overexpression of rhGas6 (Figure 3C). These results suggest that rhGas6 can promote angiogenesis both *ex vivo* and *in vivo*.

### Knockdown of *gas6* inhibits angiogenesis in zebrafish

**Figure 4:**
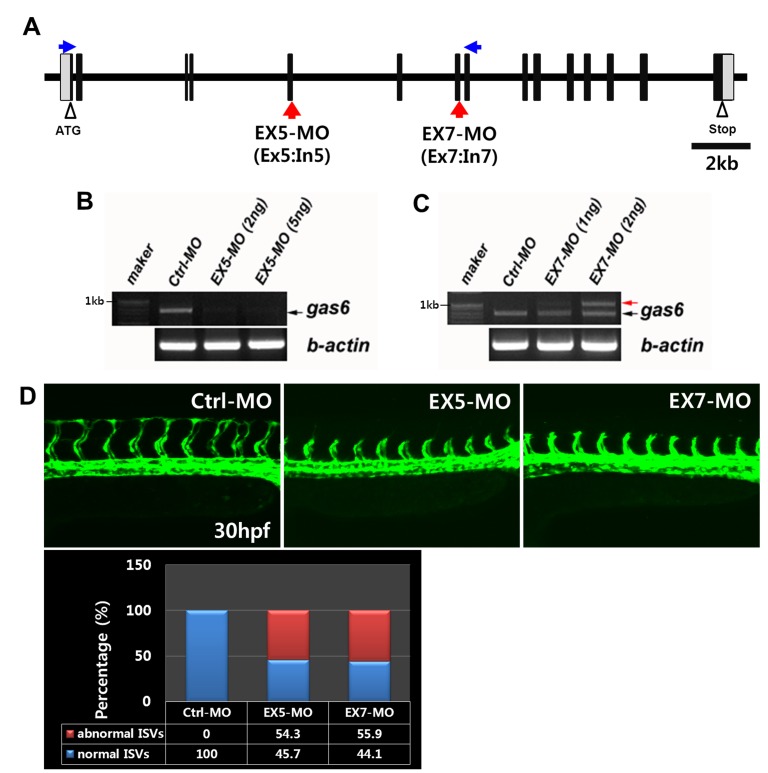
Knockdown of *gas6* induces the inhibition of angiogenesis in intersegmental vessels. (**A**) The red arrows (Ex5:In5 and Ex7:In7) indicate morpholino target sites for splicing blocks. Primers (blue arrows, exon 1, forward primer; exon 8, reverse primer) were designed for the testing of morpholino efficacy, as described in the Materials and Methods. (**B**, **C**) Testing and quantification of morpholino nucleotide efficacy by RT-PCR in standard control MO (Ctrl-MO) and *gas6* MO (EX5-MO and EX7-MO) treated embryos at 26 hpf. In control morphants, *gas6* mRNA (768 bp, black arrow) is detectable by RT-PCR (**B**, first line). In *gas6* EX5 morphants, the wild-type *gas6* mRNA is undetectable by RT-PCR at doses of 2 and 5 ng/embryo. The morphant mRNA encodes a truncated form of the Gas6 protein. (**C**) In *gas6* EX7 morphants, RT-PCR products reveal Ctrl-MO embryos expressing wild-type *gas6* transcript, while EX7-MO embryos express two transcript variants at doses of 1 or 2 ng/embryo. The black arrow shows reduced expression of wild-type *gas6* mRNA. The red arrow indicates results from aberrant splicing, resulting in a gain of ∼250 base pairs of intron 7, which encodes a premature stop codon that occurs in the *Gas6*. (**D**) Angiogenesis defects in *gas6* morphants. The flk:GFP transgenic zebrafish embryos were microinjected with Ctrl-MO (n = 30, 4 ng/embryo) and *gas6* MO (EX5-MO, n = 35, 4 ng/embryo; EX7-MO, n = 34, 2 ng/embryo), and their blood vessel formation was examined at a cellular level in living embryos at 30 hpf. Normal formation of intersegmental vessels, as shown by GFP-positive endothelial cells, is observed in Ctrl-MO embryos, but severe vascular defects is observed in *gas6* MO-injected embryos. The experiment was repeated two times.

To investigate the functional role of *gas6* in zebrafish vessel development, we performed gene knockdown studies using antisense (EX5-MO and EX7-MO). MOs were targeted against the exon 5 and exon 7 splice donor site to prevent mRNA splicing, resulting in intron insertion and thus premature translation termination (Figure 4A). We examined MOs efficacy by RT-PCR and reduced a product of *gas6* transcripts in EX5-MO injected embryos, which was not observed in control MO injected embryos (Figure 4B). We also inserted an intron 7 region by blocking of *gas6* mRNA splicing in EX7-MO injected embryos (Figure 4C). We identified the existence of intron 7 in the exon 7 morphants by sequencing of control- and *gas6*-morphant mRNA. Translation of the mRNA sequences in morphants generated a premature stop codon for the exon 5-MO and the exon 7-MO, resulting in a truncated form of the Gas6 protein. After microinjection of *gas6* MOs into zebrafish flk:GFP transgenic embryos, blood vessel formation was examined. The Ctrl-MO injected embryos exhibited normal development in the axial vasculature, dorsal aorta, posterior cardinal vein, and the dorsal longitudinal anastomotic vessel (Figure 4D, left panel). However, there were defects in intersegmental vessels and dorsal longitudinal anastomotic vessel formation in morphants at 30 hpf (Figure 4D, EX5-MO and EX7-MO). Next, we examined whether rhGas6 could restore the effect of *gas6* morphants. Injection of rhGas6 rescued the phenotypic change using microinjection of *gas6* MOs into zebrafish flk:GFP transgenic embryos (Figure S1 in File S1). Thus, these data suggest that inhibition of Gas6 signaling resulted in defects in intersegmental vessel formation in zebrafish angiogenesis.

### ERK signaling pathway in rhGas6-induced HRMECs and in *gas6*-morphants

**Figure 5:**
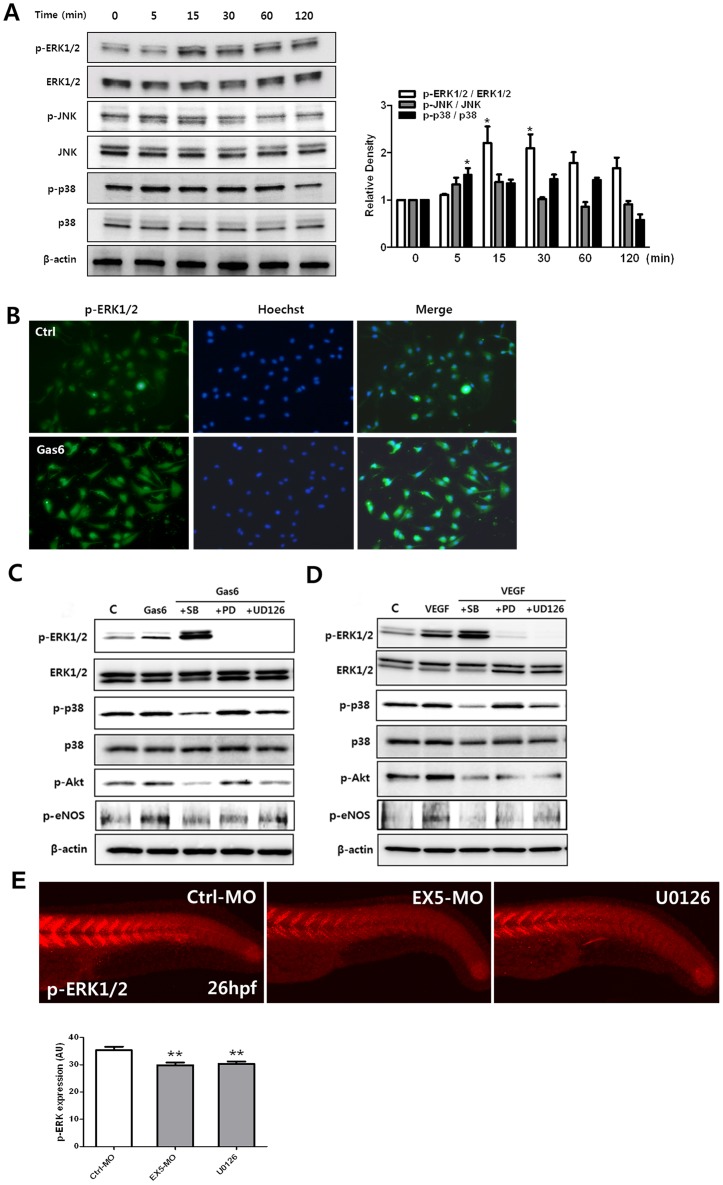
Activation of ERK is regulated by Gas6. (**A**) rhGas6 induced signaling pathway in HRMECs. Cells were incubated with rhGas6 (400 ng/ml) at various times, and cell lysates were subjected to western blotting with specific antibodies, as described in Materials and Methods. (**B**) Immunofluorescence staining for pERK1/2 and Hoechst staining was performed, as described in Materials and Methods. Representative images of p-ERK1/2 staining in rhGas6-treated HRMECs. (**C,** **D**) PD98059, SB203580, or U0126 was preincubated for 30 min, and HRMEC responses to rhGas6 or rhVEGF were determined by western blotting using specific antibodies. The experiment was repeated three times. (**E**) Phospho-ERK1/2 staining in the trunk of 26 hpf embryos [Ctrl-MO embryo (n = 7); *gas6*-MO injected embryo (n = 8); U0126-treated embryo (n = 8)] and quantitative analysis of zebrafish p-ERK1/2 expression by whole-mount immunostaining. The *gas6*-morphant and U0126 treated embryos showed reduction of p-ERK1/2 protein expression compared to controls in the zebrafish trunk. The experiment was repeated two times. **p<0.01 vs. Ctrl-MO.

To investigate the mechanism of rhGas6-induced migration in HRMECs, we examined the signal transduction pathway in rhGas6-induced HRMECs. To determine whether mitogen-activated protein kinases (MAPKs) such as ERK1/2, p38, and JNK are involved in rhGas6-induced migration of HRMECs, the phosphorylation levels of MAPKs were measured. As shown in Figure 5A, rhGas6 (400 ng/ml) treatment resulted in a transient phosphorylation of all MAPKs. The levels of phosphorylated ERK1/2 and JNK maximally increased at 15 min after Gas6 treatment, and the levels of phosphorylated p38 increased at 5 min. Gas6 levels were also increased in addition to phosphorylated ERK1/2 levels, as determined by immunofluorescence staining (Figure 5B).

Among the MAPKs, phosphorylated ERK1/2 is mainly observed during the stimulation of receptor tyrosine kinase activity by a growth factor, and is a major participant in the regulation of cell growth and differentiation. This appears to be a common and central component within various signal transduction pathways [18]. In order to determine the key signal transduction pathways involved with Gas6 induced angiogenesis in HRMEC, we evaluated the effects of MAPK inhibitors on MAPK, Akt, and eNOS signaling. Gas6 stimulated the phosphorylation of ERK1/2, p38, pAkt, and eNOS. Inhibitors SB203580 (p38), PD98059 (ERK), or U0126 (MEK/ERK) inhibited the Gas6-induced signaling (Figure 5C). The phosphorylation of ERK was associated with Gas6 induced signaling. Furthermore, to investigate the signal transduction pathways of Gas6 *in vivo*, we examined the effects of Gas6 on phosphorylation of ERK1/2 in developing zebrafish embryos. After microinjection of *gas6* MO into zebrafish embryos, we analyzed the phosphorylation of ERK1/2 as a downstream effector of Gas6 signaling. The results of whole-mount immunostaining of embryos with anti-phospho-ERK1/2 antibody revealed that repression of Gas6 reduced phosphorylation of ERK1/2 (Figure 5E, middle panel, 25% inhibition). Treatment with U0126 also reduced phosphorylation of ERK1/2 (Figure 5E, right panel, 24% inhibition).

### Migration and phenotypic changes mediated by the ERK inhibitor in Gas6-induced angiogenic processes

**Figure 6:**
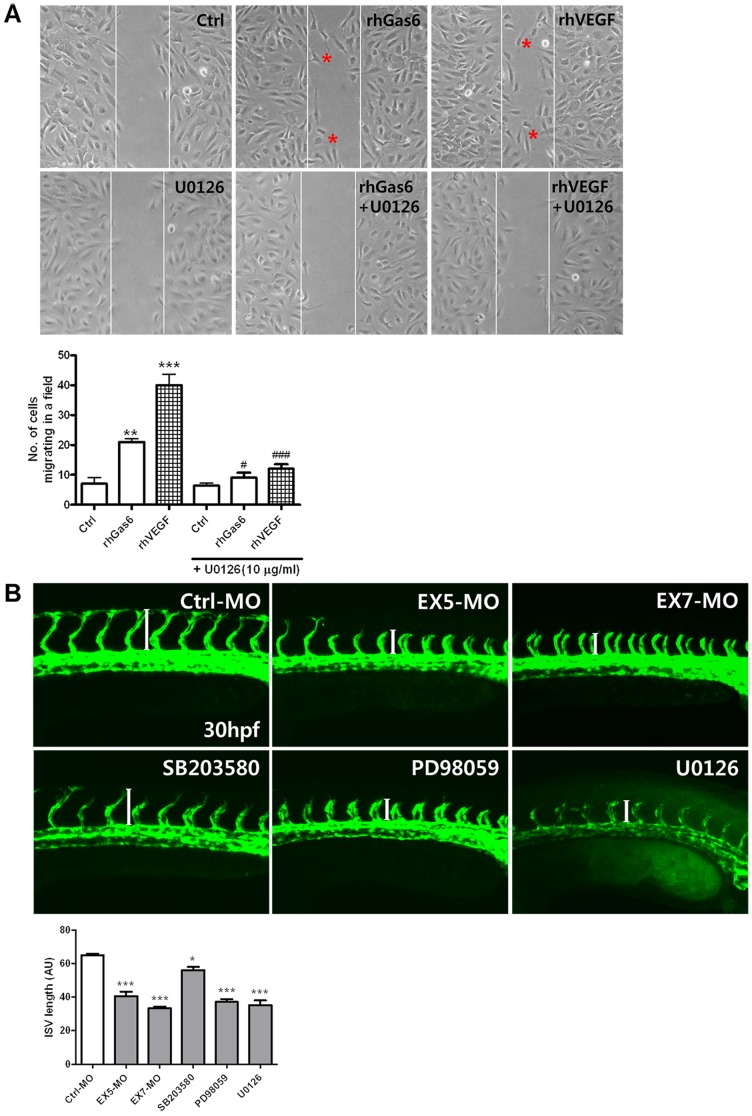
Disruption of Gas6 signaling via the ERK pathway resulted in defective angiogenesis in HRMECs and in zebrafish. (**A**) U0126 was preincubated for 30 min, and HRMEC responses to rhGas6 or rhVEGF were determined using a scratch-wound healing assay. Lines indicate the same width of the gap, and migrating cells are marked with a red asterisk. Representative images at 6 h after generating the scratch are shown. The experiment was repeated three times. ^***^ *p*<0.001, ^**^ *p*<0.01 vs. control, ^###^ *p*<0.001, ^#^ *p*<.05 vs. U0126-treated cells. (**B**) At 30 h, fluorescent images show gross morphology of Ctrl-MO injected control (n = 34), *gas6*-morpholino [EX5-MO (n = 35), EX7-MO (n = 30)] injected, and SB203580 (n = 12), or PD98059 (n = 12), or U0126 (n = 12) treated embryos. The experiment was repeated two times. Fluorescent micrographs of live flk:GFP zebrafish Ctrl-MO embryos at 30 hpf. Note the proper formation of the major axial vasculature, dorsal aorta, and posterior cardinal vein, as well as the intersegmental vessel. Representative embryos treated with *gas6*-MO oligonucleotide, SB203580, PD98059, or U0126. Note the abnormal and stunted formation of the intersegmental vessel (longitudinal white bar in **B**). Representative mild defect of intersegmental vessels in the embryos treated with SB203580. ^***^ *p*<0.001, ^*^ *p*<0.05 vs. Ctrl-MO.

To investigate the effect of the ERK1/2 inhibitor on migration of Gas6 induced HRMECs, the cells were pretreated with a MEK/ERK kinase inhibitor (U0126) and Gas6 for 6 h. For quantitating relative migration, the number of cells migrating in a field was counted. U0126 treatment significantly inhibited rhGas6 or rhVEGF induced migration (Figure 6A). Next, to test the effect of receptor tyrosine kinase inhibitors on migration of Gas6 or VEGF induced HRMECs, cells were pretreated with foretinib (0.1 µM) or SU11248 (0.1 µM) and rhGas6 or rhVEGF for 6 h. rhGas6 or rhVEGF induced the migration and the induced migration was inhibited by pretreatment with foretinib or SU11248 (Figure S2 in File S1).

To further investigate the effect of signaling molecule expression on phenotypic changes in angiogenic processes, we treated with SB203580 (p38 MAPK inhibitor), PD98059 (ERK inhibitor), or U0126 (MEK/ERK inhibitor), and injected EX5-MO and EX7-MO into flk:GFP transgenic zebrafish embryos using previously described methods. In this transgenic system, endothelial cells can be directly observed under a fluorescence stereomicroscope. The specificity of the MEK/ERK inhibitor has already been reported *in vivo* [19], including analysis in zebrafish [8], [9]. As shown in Figure 6B, treatment with inhibitors (SB203580, PD98059, or U0126) at 12 hpf inhibited intersegmental vessel formation compared to control levels (treated with 0.4% DMSO alone) at 30 hpf. Similar results were observed with knockdown of *gas6* (EX5-MO and EX7-MO) in embryos (Figure 6B). These results suggest that Gas6 can regulate angiogenesis by activation of the MEK/ERK kinase pathway.

## Discussion

Gas6 has been shown to play a pivotal role in pathophysiological processes such as atherosclerosis, cancer, and thrombosis through activation of cells ranging from platelets to endothelial cells, as well as vascular smooth muscle cells [6]. Gas6 is secreted or expressed by various cancer cells, smooth muscle cells, retinal pigment epithelial cells, mesangial cells, and endothelial cells [7], [9], [20]. Expression of Gas6 and Axl is increased in various types of cancers, and Axl has also been suggested as a rational target for cancer therapy [21], [22]. In the retina, Gas6 is expressed endogenously by human retinal pigment epithelial cells, and deficiency or inhibition of Gas6 induces platelet dysfunction and protects from thrombosis [6], [23]. However, there has been no study on the effect of Gas6 on the functions and mechanisms mediated by HRMECs. The results from this study provide evidence that Gas6 stimulates angiogenesis involving proliferation, migration, and sprouting of endothelial cells and of zebrafish embryos via ERK1/2 signaling.

Angiogenesis, a process of new blood vessel growth, is induced by various growth factors such as VEGF, FGF, PDGF, and transforming growth factor-beta (TGF-β), and it is a target for combating diseases characterized by either poor vascularization or abnormal vasculature. Pathological angiogenesis in the retina is a major feature of diseases that lead to blindness, particularly diabetic retinopathy [24]. Inhibiting angiogenesis by targeting specific pro-angiogenic factors such as VEGF has become a major focus of drug development for diabetic retinopathy, and anti-VEGF drugs are used to treat diabetic retinopathy [25], [26]. The mechanism of action by which Gas6 induces neovascularization in retinal microendothelial cells is still subject to speculation. In this study, Gas6 promoted the *in vitro* proliferation of HRMECs in a time- and dose-dependent manner (Figure 1). Gas6 also induced migration and tube formation in HRMECs. Furthermore, vessel sprouting on the rat aorta was stimulated by rhGas6, and the outgrowth of endothelial tubules and vessel sprouting length was increased. Knockdown of *gas6* using antisense MOs inhibited angiogenesis in zebrafish development (Figure 4). To confirm that the phenotypes observed in gas6 MO-injected embryos were caused by loss of function in gas6, we injected MO and rhGas6 protein. rhGas6 could partially rescue of gas6 MO-induced angiogenesis defect at 30 hpf, suggesting that the defect caused by injecting the gas6 MO was induced caused by a knockdown of gas6 activity (Figure S1). Gas6 protein has been shown to interact with receptor tyrosine kinases Axl, Mer, and Tyro3 [27]. To effectively link Axl, Mer, and Tyro3 expression levels to specific cellular behaviors in HRMECs, we used a collection of Axl, Mer, and Tyro3 targeting shRNAs that reduced Axl, Mer, and Tyro3 expressions in a graded manner. Axl, Mer, or Tyro3 shRNA transfected cells showed reduction of Axl, Mer, or Tyro3 mRNA levels, and Gas6 induced Axl, Mer, or Tyro3 mRNAs levels (Figure S3 in File S1). Moreover, inhibitors of receptor tyrosine kinases inhibited rhGas6 induced migration (Figure S2 in File S1). Gas6 induced signaling pathway effects on migration may therefore mediate the receptor tyrosine kinase family members, Axl, Mer, and Tyro3, in HRMECs.

Prevention of neovessel growth is a promising strategy of intervention to improve long-term prognosis of visual outcome and quality of life for many patients. Cell migration is essential in pathophysiological processes, such as wound healing and metastasis. VEGF induces neovessel growth and migration in the development of both proliferative diabetic retinopathy and diabetic macular edema, and anti-VEGF agents have emerged as new approaches in the treatment of these devastating diabetic complications [28]. The Ras-dependent ERK1/2 MAP kinase pathway plays a central role in cell proliferation control [29]. Recently, it was shown that inhibition of ERK activation, which occurs immediately after wounding, significantly inhibited the directional migration of fibroblasts [30]. ERK1/2 inhibitors and *gas6* MOs reduced phosphorylation of ERK1/2 in zebrafish, and phenotypic changes were also significantly inhibited by *gas6* MOs microinjection into transgenic (flk:GFP) zebrafish embryos. This suggests that Gas6 signaling regulates angiogenesis through ERK1/2 kinase.

To our knowledge, this study provides the first evidence that Gas6 can induce proliferation, migration, and angiogenesis in HRMECs and in zebrafish during vessel formation. Moreover, these processes may occur via phosphorylation of ERK1/2.

## Supporting Information

File S1 **Contains the files: Figure S1. Recombinant human Gas6 rescues the phenotypic changes in** ***gas6*** **morphant-injected embryos.** (**A**) Embryos injected with *gas6*-exon5 morpholino. (**B**) Embryos injected with *gas6*-exon7 morpholino. Shown above are 30 hpf zebrafish Ctrl-MO-injected embryos (Ctrl-MO), embryos injected with a *gas6* morpholino (EX-5 MO, EX-7 MO), injected with a *gas6* morpholino and rhGas6 protein [EX-5 MO + rhGas6 (130 ng/µl), EX-7 MO + rhGas6 (130 ng/µl)]. Injection of rhGas6 resulted in a rescue of ISVs defects. ISVs formation is indicated by the white dotted line. **Figure S2.** **Effect of receptor tyrosine kinase inhibitors on rhGas6-induced migration in HRMECs.** Wound-healing cell migration assay was performed as described in Material and Methods. Foretinib and SU11248 were preincubated for 30 min, and Gas6 was then incubated for 6 h in the HRMECs. Foretinib and SU11248 inhibit Gas6 induced migration in HRMECs. **Figure S3.** **rhGas6 stimulated the expression of Axl, Mer, and Tyro3 in HRMECs.** The HRMECs were transfected with Axl, Mer, or Tyro3 shRNA and treated with rhGas6 (400 ng/ml). shRNA knockdown and quantitative real-time PCR were performed as described in Material and Methods. Gas6 induced Axl, Mer, and Tyro3 mRNA expression. Each bar represents the mean ± SD from three independent experiments (***p<0.001 vs. control shRNA; ###*p*<0.001 vs. rhGas6-untreated and control shRNA-treated cells).
(DOCX)
