# Supplementary figures and images for: Gas6 Stimulates Angiogenesis of Human Retinal Endothelial Cells and of Zebrafish Embryos via ERK1/2 Signaling
Source: PLoS One. 2014 Jan 7;9(1):e83901. doi: 10.1371/journal.pone.0083901 (PMC3883657; doi:10.1371/journal.pone.0083901)

**Figure S1.**

**
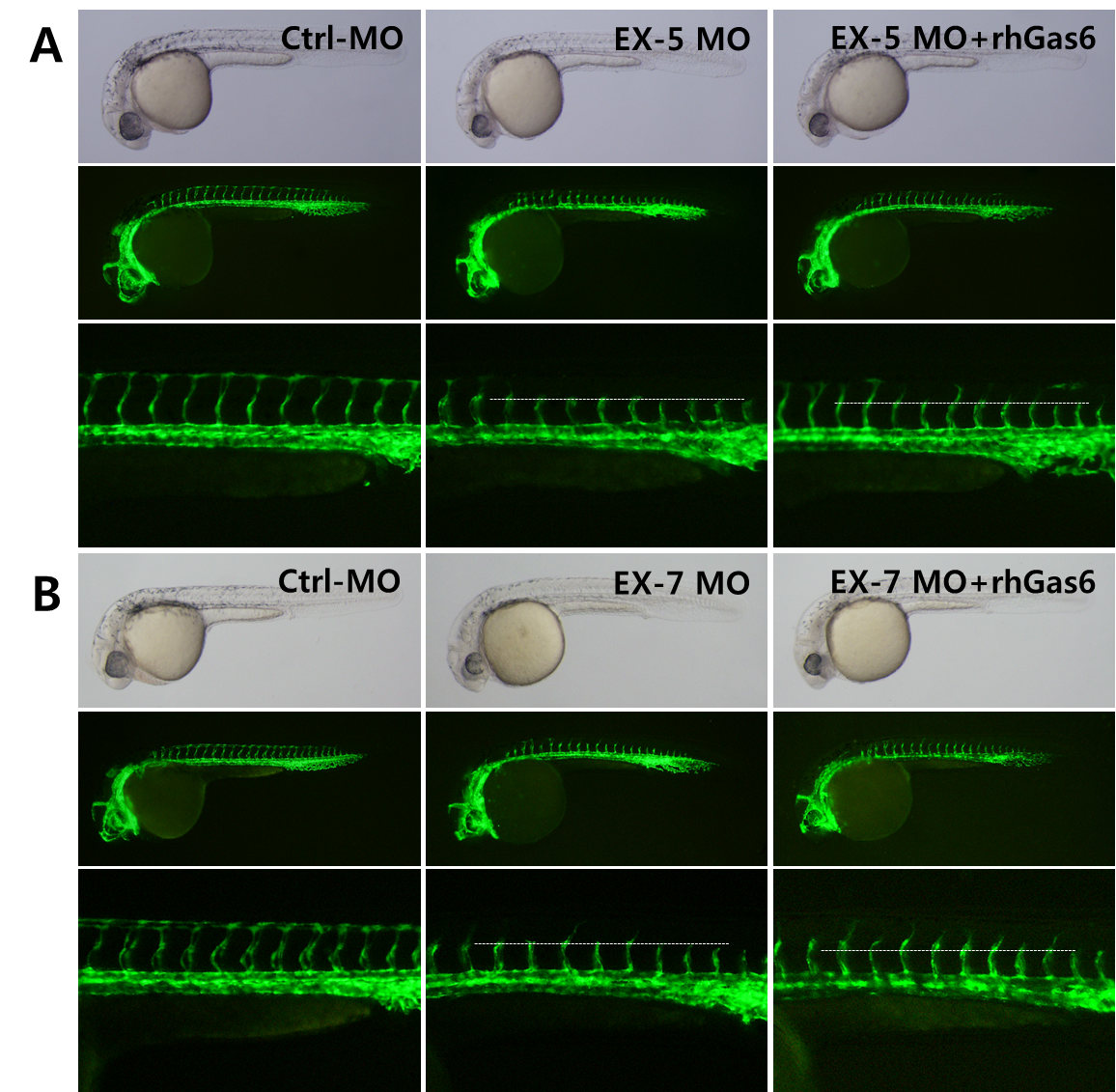
**

**Figure S2.**


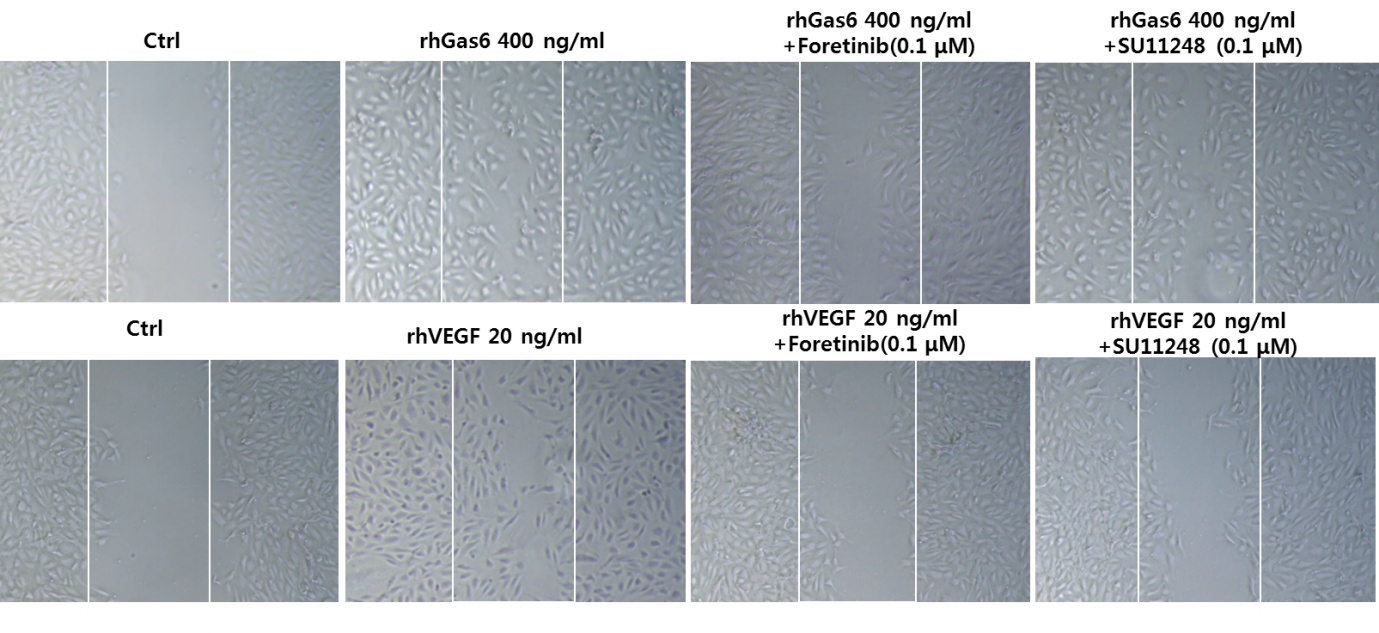


**Figure S3.**

Supplement: File S1 — Contains the files: Figure S1. Recombinant human Gas6 rescues the phenotypic changes in gas6 morphant-injected embryos. (A) Embryos injected with gas6-exon5 morpholino. (B) Embryos injected with gas6-exon7 morpholino. Shown above are 30 hpf zebrafish Ctrl-MO-injected embryos (Ctrl-MO), embryos injected with a gas6 morpholino (EX-5 MO, EX-7 MO), injected with a gas6 morpholino and rhGas6 protein [EX-5 MO + rhGas6 (130 ng/µl), EX-7 MO + rhGas6 (130 ng/µl)]. Injection of rhGas6 resulted in a rescue of ISVs defects. ISVs formation is indicated by the white dotted line. Figure S2. Effect of receptor tyrosine kinase inhibitors on rhGas6-induced migration in HRMECs. Wound-healing cell migration assay was performed as described in Material and Methods. Foretinib and SU11248 were preincubated for 30 min, and Gas6 was then incubated for 6 h in the HRMECs. Foretinib and SU11248 inhibit Gas6 induced migration in HRMECs. Figure S3. rhGas6 stimulated the expression of Axl, Mer, and Tyro3 in HRMECs. The HRMECs were transfected with Axl, Mer, or Tyro3 shRNA and treated with rhGas6 (400 ng/ml). shRNA knockdown and quantitative real-time PCR were performed as described in Material and Methods. Gas6 induced Axl, Mer, and Tyro3 mRNA expression. Each bar represents the mean ± SD from three independent experiments (***p<0.001 vs. control shRNA; ###p<0.001 vs. rhGas6-untreated and control shRNA-treated cells). (DOCX) [file pone.0083901.s001.docx]
